# Short-Term Effects of Temperature and Thyrotropin-Releasing Hormone Stimulation on Adrenocorticotropin Stability in Horses

**DOI:** 10.3390/ani12030324

**Published:** 2022-01-28

**Authors:** Sophia L. Hinrichsen, Ka Y. Yuen, Elizabeth L. Dryburgh, François-René Bertin, Allison J. Stewart

**Affiliations:** 1 School of Veterinary Science, The University of Queensland, Gatton, QLD 4343, Australia; sophia.hinrichsen@uq.net.au (S.L.H.); k.yuen@uq.edu.au (K.Y.Y.); f.bertin@uq.edu.au (F.-R.B.); 2 Boehringer Ingelheim Animal Health Australia Pty. Ltd., North Ryde, NSW 2113, Australia; liz.dryburgh@boehringer-ingelheim.com

**Keywords:** endocrinology, pituitary pars intermedia dysfunction, chemiluminescent assay, geriatric

## Abstract

**Simple Summary:**

Approximately 20% of older horses develop pituitary dysfunction (PPID), which is associated with haircoat changes, muscle loss, and a higher risk of developing an infection or laminitis. Elevated plasma adrenocorticotropic hormone (ACTH) is used to diagnose PPID; however, ACTH is not stable in blood samples. Therefore, samples should be kept at 4 °C until analysis. In ambulatory veterinary practice, blood samples can be left at room temperature (20 or 30 °C) or inadvertently left in a vehicle without refrigeration where they might be exposed to temperatures of up to 70 °C in hot climates. To evaluate the effects of temperature on ACTH concentrations, we experimentally subjected blood samples from horses with and without pituitary dysfunction to temperatures of 4 (reference), 20, 30, and 70 °C for 1 h prior to laboratory measurement. The stability of ACTH was affected by short-term exposure to high temperatures in horses with and without pituitary dysfunction with both higher and lower ACTH concentrations measured unpredictably. Our results suggest that samples should be kept at 4 °C to reflect the true ACTH concentration. Exposure to temperatures of up to 40 °C for 1 h can still provide an appropriate assessment of pituitary function in most cases, but the ACTH concentration changed by 12% in healthy horses and 5% in horses with PPID.

**Abstract:**

Pituitary pars intermedia dysfunction (PPID) is diagnosed by increased basal or post thyrotropin-releasing hormone (TRH) stimulation ACTH concentrations. ACTH is known to be unstable; however, the effect of different temperatures and TRH stimulation on equine ACTH stability is poorly described. In total, 15 horses, including 8 PPID positive (ACTH > 35 pg/mL at baseline or >65 pg/mL 30 min after TRH stimulation), were divided into 2 groups: 9, including 5 PPID positive, with basal ACTH concentrations and 6, including 3 PPID positive, with post TRH stimulation ACTH concentrations. Whole blood was stored for 1 h at 4, 20, 30, 40, or 70 °C. After centrifugation, immunoreactive ACTH concentrations were determined using a chemiluminescent assay. Linear mixed effect models were used to detect the effects of temperature, PPID status, and TRH stimulation on the immunoreactive ACTH concentration. Temperature had a significant effect (*p* = 0.003) on immunoreactive ACTH concentrations, and this effect was greater in PPID-negative horses (*p* = 0.01), with the changes in immunoreactive ACTH concentrations being slightly unpredictably higher or lower than samples stored at 4 °C. Even at 20 °C, mean immunoreactive ACTH concentrations minimally changed by 5% in PPID horses and 12% in non-PPID horses after 1 h. No significant effect of TRH stimulation was identified. Although ACTH concentrations should ideally be determined from samples kept at 4 °C, samples inadvertently left at temperatures of up to 40 °C can provide valid results if analyzed within 1 h; however, this increases the risks of altered ACTH concentrations, occasionally influencing the diagnosis of PPID.

## 1. Introduction

Pituitary pars intermedia dysfunction (PPID) is a common disease of older horses and ponies, affecting 21% of horses over 15 years of age [1,2]. Up to 73% of PPID-positive horses are euthanized due to complications associated with PPID and only 50% of horses are alive 4.5 years after diagnosis [3].

Histologic examination of the pars intermedia is considered the gold standard to diagnose PPID; however, there is only fair agreement between pathologists, and histopathology can only be performed postmortem [4,5]. Recommended antemortem diagnostic tests include baseline ACTH concentration and ACTH response to thyrotropin-releasing hormone (TRH) stimulation, which increases diagnostic test sensitivity to diagnose early or subclinical cases of PPID [6,7,8,9,10].

Studies found that human ACTH is highly unstable due to proteolytic degradation [11,12]. Preanalytical stability of human ACTH depends on both the time to centrifugation and temperature, with the measured ACTH concentration being altered if blood samples are left at room temperature for ≥4 h prior to centrifugation. In order to obtain accurate values of plasma ACTH concentrations in humans, it has been recommended that if samples cannot be transported to a laboratory for analysis within 2 h (at room temperature), they should be kept at 4 °C and analyzed within 8 h [13]. The effects on the measured ACTH concentration of delaying centrifugation of horse blood by even 1 h resulted in a mean reduction in the measured ACTH concentration by 11.6 pg/mL in one study [14]. The stability of equine ACTH has been investigated at room temperature (20 or 22 °C) and with archived samples (−20 or −80 °C), but as far as the investigators are aware, there have been no studies investigating the stability of ACTH when exposed to high environmental temperatures [15,16]. Some countries or regions are susceptible to high environmental temperatures and samples obtained by equine ambulatory veterinarians might be subjected to these conditions if not kept in adequate cooling devices. In some areas, environmental temperatures might occasionally exceed 40 °C and temperatures inside vehicles might exceed 70 °C [17,18]. It is also uncertain if ACTH post-TRH stimulation has the same stability as basal ACTH. Previous research could not detect an effect of TRH stimulation on the stability of equine immunoreactive ACTH after up to five freeze/thaw cycles, suggesting that ACTH post-TRH stimulation would have a similar stability to basal ACTH; however, further investigation is warranted to determine if there is an effect of TRH stimulation on the stability of ACTH after exposure to high temperatures [18].

If inappropriate sample handling conditions affect the measured ACTH concentration, then false positive and negative diagnoses of PPID might occur. This could have animal welfare implications if the ACTH concentration is reduced, and a false negative result is obtained [6]. In contrast, a false positive result could lead to unnecessary medication and might even prevent animals from competing in high-level competitions where pergolide is a banned substance (FEI Equine Prohibited Substances List, January 2020).

Therefore, the aim of this study was to investigate the short-term effect of a range of temperatures, and the effect of TRH stimulation, on ACTH stability in horses with and without PPID, and the effect on the subsequent diagnosis of PPID.

## 2. Materials and Methods

### 2.1. Horses

In total, 15 institution-owned mature horses (median 16 years of age, range 11 to 27 years) were enrolled. There were 6 geldings and 9 mares of various breeds: Australian Stock Horse (*n* = 6), Standardbred (*n* = 5), Warmblood (*n* = 2), Arabian (*n* = 1), and Quarter Horse (*n* = 1). Horses were kept on the same pasture. In total, 8 horses were diagnosed with PPID in mid-summer (December and January) by either a baseline ACTH concentration over 35 pg/mL or ACTH concentration over 65 pg/mL 30 min post-TRH stimulation [8,9,10,19]. All horses assigned to the PPID-positive group either had clinical signs or developed them during the following 12 months. Beyond clinical signs of PPID for some horses, all were healthy based on physical examination [20]. No horses were receiving any medication including pergolide.

Horses were randomly divided (coin toss) into two groups. Baseline ACTH was determined in 6 horses, of which 3 were PPID positive. The second group included 9 horses, of which 5 were PPID positive, and ACTH concentrations were determined 30 min post-TRH stimulation.

### 2.2. Sample Processing

For horses in which the basal ACTH concentration was determined, 50 mL of blood were collected by jugular venipuncture into 5 ethylenediaminetetraacetic acid (EDTA) plastic blood collection tubes from each horse. For horses that underwent TRH stimulation, blood was collected 30 min after receiving 1 mg of TRH intravenously (Sigma-Aldrich Pty Ltd. (subsidiary of Merck), North Ryde BC, New South Wales, Australia). Blood was sampled between 7 and 8 am. All procedures were approved by the University of Queensland Animal Ethics Committee, approval number SVS/474/17, 22nd December 2017.

Samples were taken to the laboratory within 1 h of collection. They were then stored as whole blood either in a temperature-monitored refrigerator (4 °C), in a temperature-controlled room (20 °C), or in incubators (30, 40, or 70 °C) for 1 h. Samples were then centrifuged at 3500× *g* at 4 °C for 10 min. The plasma was separated and analyzed within 2 h of collection from the horse. The plasma immunoreactive ACTH concentration was measured by a chemiluminescent assay (Immulite 1000 Chemiluminescent Assay, Siemens. Bayswater, VIC 3153, Australia). The intra-assay coefficient of variation used for this assay was 4.8% [18]. Samples were analyzed using kits from the same ACTH lot numbers that had been purchased and transported to our laboratory as a single acquisition. Due to the necessity of analyzing all samples within a 2-h window from collection, and limitations on the speed of the analyzer, the 15 horses were sampled over a 4-day period. The samples from each horse were run using the same kit.

### 2.3. Data Analysis

To satisfy distribution normality as tested with the Kolmogorov–Smirnov test, the changes in the immunoreactive ACTH concentrations in each sample were calculated utilizing both the percentage of the reference sample (ACTH concentration in sample/ACTH concentration in reference sample × 100) and the absolute percentage change from the reference sample (absolute value of (1-(ACTH concentration in sample/ACTH concentration in reference sample)) × 100). The reference sample was defined as the sample stored at 4 °C. The percentage of the reference sample (presented as mean ± standard deviation) was the percent deviation above or below the ACTH concentration of the reference sample (recorded as 100%) while the absolute percentage change from the reference sample (presented as mean ± standard deviation) was used to show an absolute value that deviated away from the ACTH concentration of the reference sample (recorded as 0%).

The effects of temperature, TRH stimulation, and PPID status were then analyzed with a linear mixed effect model using “temperature”, “TRH stimulation”, and “PPID status” as fixed effects and “horse” as a random effect to account for repeated measures. Then, concentrations of immunoreactive ACTH were compared to baseline (expressed as either 100% or 0%) using a repeated measures ANOVA and Tukey’s multiple comparison’s test. The statistical analysis was performed by commercially available software (IBM^®^ SPSS^®^ Statistics Version 25) and *p* < 0.05 was considered to be significant.

To determine the effects of short-term high-temperature exposure on a diagnosis of PPID, a qualitative analysis was performed for each sample, with a cut-off value of >35 pg/mL in the horses in which the basal ACTH concentration was determined, or >65 pg/mL in the horses in which the post-TRH stimulation test ACTH concentration was determined. A false positive and false negative rate was then determined for each temperature.

Bland–Altman plots were used to determine the bias (95% limits of agreement (LOA)) and visualize the difference between ACTH concentrations in samples kept at 4 °C versus 20, 30, 40, and 70 °C for 1 h.

## 3. Results

**Figure 1:**
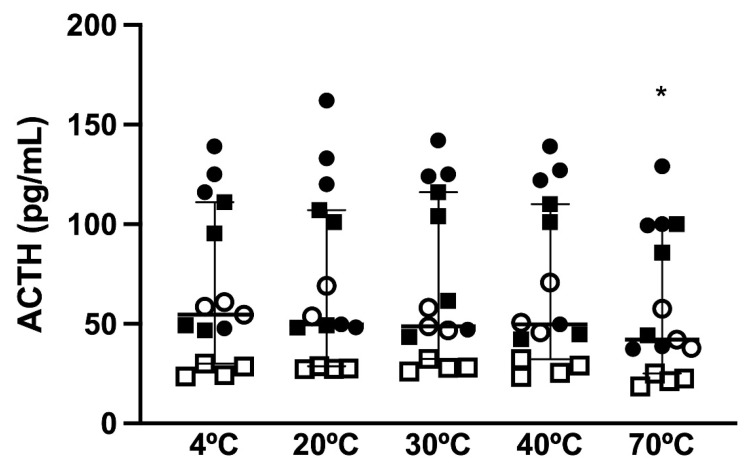
ACTH concentrations in samples kept at 4 (reference sample), 20, 30, 40, and 70 °C in 15 horses (median and 95% CI). Squares represent horses in which the basal ACTH concentration was determined and circles indicate horses in which the post-TRH stimulation ACTH concentration was determined. Hollow shapes represent non-PPID horses and solid shapes indicate horses with PPID. * *p* < 0.0003 for 4° vs. 70 °C.

**Figure 2:**
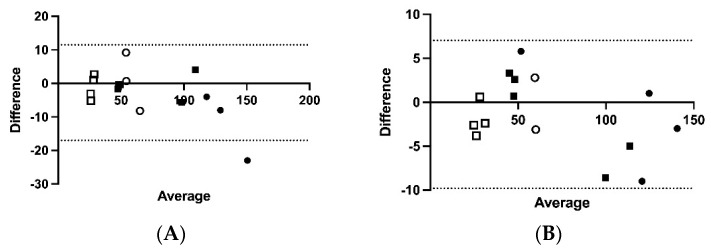

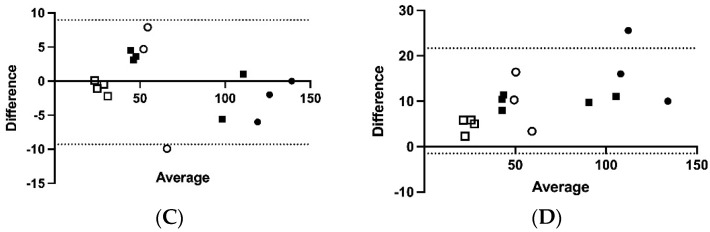
Bland–Altman plots showing the difference in ACTH concentrations (pg/mL) in samples kept at: (**A**) 4 °C versus 20 °C; (**B**) 4 °C versus 30 °C; (**C**) 4 °C versus 40 °C; and (**D**) 4 °C versus 70 °C in 15 horses. The solid lines represent the mean differences and the dotted lines represent the 95% level of agreement. Squares represent horses in which the basal ACTH concentration was determined and circles indicate horses in which the post-TRH stimulation ACTH concentration was determined. Hollow shapes represent non-PPID horses and solid shapes indicate horses with PPID.

There was a difference in the effects of short-term storage temperature (*p* < 0.0001) on immunoreactive ACTH concentrations, with a difference between storage at 4 °C and storage at 70 °C (*p* = 0.0003; Figure 1). Bland–Altman plots (Figure 2A–D) demonstrated mean bias of −2.8 pg/mL [−17.04–11.46 pg/mL] for 4 versus 20 °C, −1.38 pg/mL (−10.0 to 7.0 pg/mL) for 4 versus 30 °C, −0.16 pg/mL (−9.3 to 9.0 pg/mL) for 4 versus 40 °C, and 10.1 pg/mL (−1.5 to 21.7 pg/mL) for 4 versus 70 °C for 1 h.

**Figure 3:**
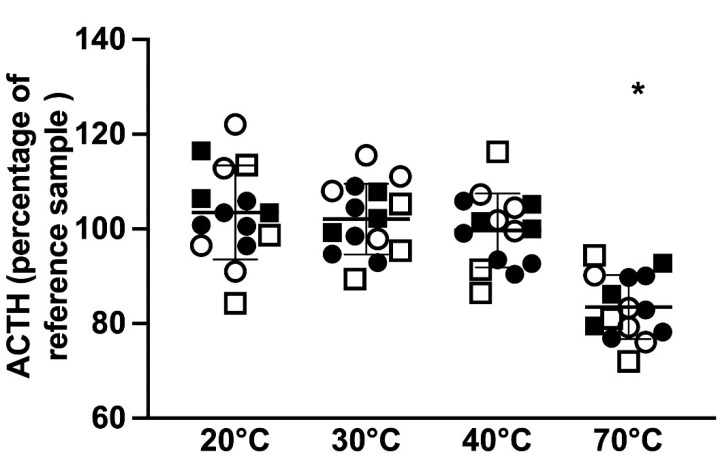
ACTH concentrations presented as a percentage of the reference sample in samples kept at 4 (reference sample), 20, 30, 40, and 70 °C in 15 horses (mean ± standard deviation). Squares represent horses in which the basal ACTH concentration was determined and circles indicate horses in which the post-TRH stimulation ACTH concentration was determined. Hollow shapes represent non-PPID horses and solid shapes indicate horses with PPID. * *p* < 0.05 vs. 4 °C.

Immunoreactive ACTH concentrations analyzed as a percentage of the reference 4 °C sample showed that only temperature had a statistically significant effect on the measured immunoreactive ACTH concentration (*p* = 0.003) and there was no significant effect of PPID status or TRH stimulation on the measured ACTH concentration. Samples kept at 20, 30, and 40 °C all showed deviations above and below the ACTH concentration of the reference sample, ranging from 84.3 to 122.1% in the samples kept at 20 °C, from 89.4 to 115.6% in the samples kept at 30 °C, and from 86.5 to 116.3% in the samples kept at 40 °C; however, none of these changes were statistically significant. Significantly lower immunoreactive ACTH concentrations were observed in samples that were kept at 70 °C, with all ACTH concentrations below the ACTH concentration of the reference sample, ranging from 72 to 94.4% (*p* = 0.001, Figure 3). However, out of the 15 samples that were kept at 70 °C for 1 h, only 9 could be analyzed due to solidification of the sample.

**Figure 4:**
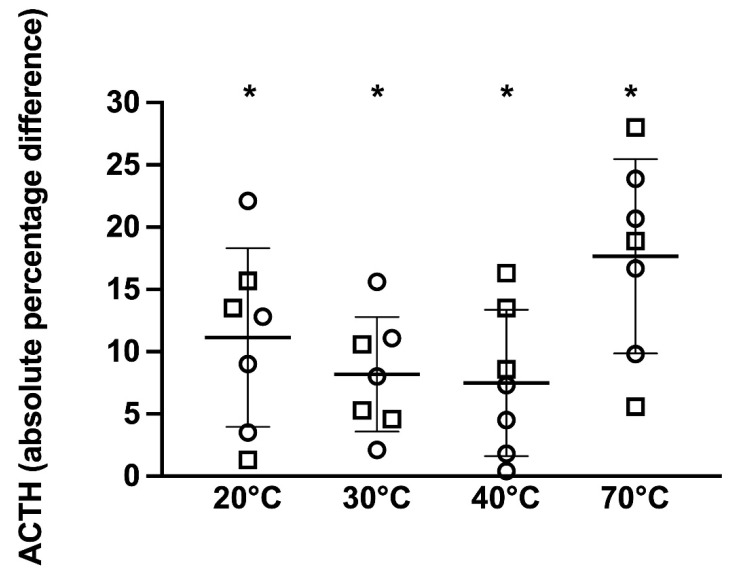
ACTH concentrations presented as the absolute percentage change from the reference sample in samples kept at 4 (reference sample), 20, 30, 40, and 70 °C in 7 non-PPID horses (hollow shapes) with mean ± standard deviation shown. Squares represent horses in which the basal ACTH concentration was determined and circles indicate horses in which the post-TRH stimulation ACTH concentration was determined. * *p* < 0.05 vs. 4 °C.

Immunoreactive ACTH concentrations analyzed as the absolute percentage change from the reference sample showed that both temperature and PPID status had a significant effect on the immunoreactive ACTH concentration (*p* = 0.001 and *p* = 0.01, respectively); however, there was no significant effect of TRH stimulation on the ACTH concentration (*p* = 0.8). For the 7 non-PPID horses, when compared to the 4 °C reference sample, samples kept at 20, 30, 40, and 70 °C had significantly different ACTH concentrations (*p* = 0.02, 0.01, 0.04, and *p* = 0.003, respectively) ranging from 1.3 to 22.1%, 2.1 to 15.6%, 0.4 to 16.3%, and 5.6 to 28%, respectively, above or below the reference sample (Figure 4).

**Figure 5:**
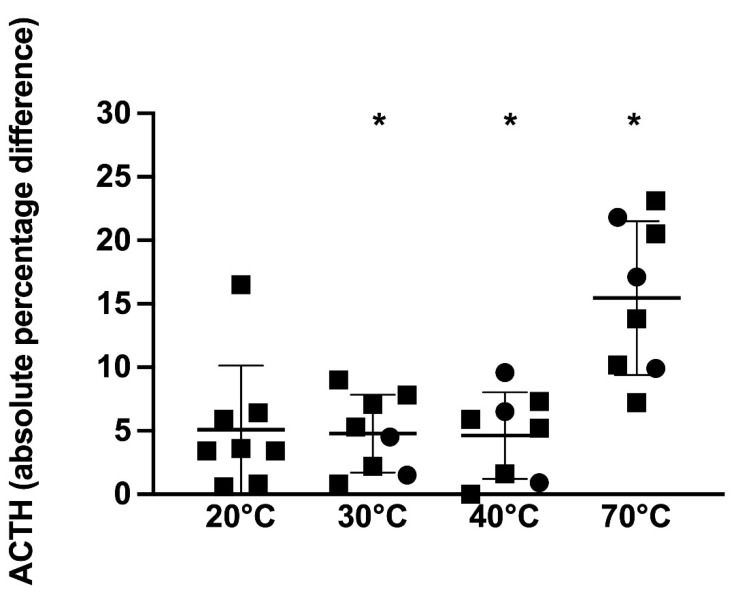
ACTH concentrations presented as the absolute percentage change from the reference sample in samples kept at 4 (reference sample), 20, 30, 40, and 70 °C in 8 PPID horses with solid shapes (mean ± standard deviation). Squares represent horses in which the basal ACTH concentration was determined and circles indicate horses in which the post-TRH stimulation ACTH concentration was determined. * *p* < 0.05 vs. 4 °C.

For the 8 PPID horses, when compared to the 4 °C reference sample, samples kept at 30, 40, and 70 °C had significantly different ACTH concentrations (*p* = 0.01, 0.02, and *p* = 0.001, respectively) ranging from 8 to 9.0%, 0 to 9.6%, and 7.2 to 23.1%, respectively, above or below the reference sample (Figure 5). No significant difference in the immunoreactive ACTH concentration was detected between samples kept at 20 °C compared to samples kept at 4 °C (*p* = 0.07), with differences ranging from 0.6 to 16.5% above or below the reference sample.

Although the immunoreactive ACTH concentrations significantly changed with temperature and PPID status, the change in the ACTH concentration was only clinically relevant in one horse, a non-PPID horse that underwent TRH stimulation. This horse had a reference 4 °C ACTH concentration of 60.9 pg/mL, but after storage of the whole blood sample at 20 and 40 °C for 1 h, the measured ACTH concentrations were 69.1 and 70.8 pg/mL, respectively. This would have led to a false positive diagnosis of PPID if a diagnostic cut-off value of 65 pg/mL was used.

## 4. Discussion

The main results of this study are that (1) statistically different changes in the measured immunoreactive ACTH concentration occurred when samples were stored as whole blood at 70 °C for as little as 1 h, (2) clinically significant changes were observed for the measured immunoreactive ACTH concentration in 1 of 15 horses when samples were stored at 20 and 40 °C, (3) the stability of immunoreactive ACTH from PPID and non-PPID horses was different, and (4) no significant effect of TRH stimulation on immunoreactive ACTH stability was detected.

Previous research has investigated the effects of ACTH stability at room temperature (21 °C) on ACTH concentration, finding no difference between samples kept at room temperature verses those kept at 4 °C when analyzed prior to 8 h [15]. After that time, the ACTH concentration of samples stored at room temperature began to decline, compared to refrigerated samples; however, it should also be noted that those results were mostly from PPID-negative horses [15]. These findings suggested that the storage of samples at room temperature could provide valid results. In contrast, our study found that samples kept at 20 °C for only 1 h led to deviations in the measured ACTH concentration of up to 22% above and 18% below the ACTH concentration measured in the same sample stored at 4 °C, suggesting that samples kept at 20 °C could potentially lead to occasional misdiagnoses. The Bland–Altman plots suggest that the bias is small for a given ACTH value at 20, 30, and 40 °C, with reasonably small increases in the 95% LOA as the temperatures increased to 40 °C. Therefore, accidently keeping a sample at room temperature, or in a pocket or car up to 40 °C for 1 h is unlikely to be clinically relevant in most cases. Leaving a sample in a car in the sun on an extremely hot day where vehicle temperatures can reach 70 °C is not recommended, with reductions in the ACTH concentration in samples that did not solidify.

We also found that individual horses with and without PPID had small random and unpredictable rises or declines in the measured immunoreactive ACTH concentration in response to short-term exposure to high temperatures (>20 °C). Horses without PPID showed more variability in the absolute percentage change in the ACTH concentration compared to horses with PPID, but their reference values at 4 °C were lower so the percentage change was higher. A previous study investigating the effect of processing delays on ACTH concentrations also found that the stability of ACTH was affected by the PPID status of the horse [16].

The differences in stability observed between PPID-positive and PPID-negative horses could be due to the site of ACTH production. PPID-negative horses release the majority of their stress-induced ACTH from the corticotropes of the pars distalis within the pituitary gland, and only a small amount from the pars intermedia [21]. In contrast, PPID-positive horses release more proopiomelanocortin (POMC)-derived peptides, such as α-melanocyte-stimulating hormone (α-MSH), corticotrophin-like intermediate peptide (CLIP), β-endorphins, and ACTH, from the melanotropes within the pars intermedia of the pituitary gland. These peptides likely have different structures than pars distalis-derived ACTH [22]. In both PPID-negative and -positive horses, prohormone convertase 2 (PC2) cleaves ACTH into α-MSH and CLIP [18,22]. It is likely the Immulite™ assay also cross reacts some of these POMC peptides. It is possible that degradation of ACTH to CLIP occurs in vitro. Horses with PPID have been shown to have an upregulated expression of the messenger ribonucleic acid of PC2 [22]. This mutation reduces the conversion of ACTH to α-MSH, resulting in high plasma concentrations of an ACTH peptide with poor bioactivity [18,22]. These ACTH peptides could be increasingly more susceptible to proteolytic degradation or have a structure that interacts differently with the antibodies used in chemiluminescent assay, resulting in variable immunoreactive ACTH concentrations [18].

It has been shown that the peptides measured as basal ACTH are different to the ACTH fragments measured after TRH stimulation, with marked differences in peptide detection using different analyzers [23]. The Immulite™ 1000 is a chemiluminescent immunometric assay that uses two-site sequential mouse monoclonal and rabbit polyclonal anti-human ACTH antibodies. The Immulite™ 1000 assay consistently measures higher values of the ACTH concentration when compared to an immunofluorescence assay in samples concurrently measured from ponies or horses for basal and TRH-stimulated ACTH [23,24,25]. Poor agreement between the Immulite 1000 and a commercial ACTH radioimmunoassay has also been documented [26]. The season also affects basal and post TRH ACTH concentrations in horses with and without PPID, and seasonally specific thresholds for the diagnosis of PPID specific for each type of analyzer should always be utilized [8,27,28]. It was unknown if post-TRH stimulation ACTH would have the same stability as basal ACTH. We were unable to detect a difference in the effects of TRH stimulation on ACTH stability, suggesting that both basal ACTH and ACTH post TRH stimulation have equal stability.

A limitation of our study was the small sample size. However, by separating the eight PPID-positive and the seven PPID-negative horses, the variability in the ACTH concentration was reduced compared with other studies [16]. Significant effects of temperature on ACTH stability were detected even when using small sample sizes, indicating adequate power of the study when absolute values were utilized. Although storage at 70 °C caused a uniform reduction in the measured ACTH concentrations, storage at temperatures between 20 and 40 °C caused some ACTH measurements to increase and some to decrease in an unpredictable manner, which was more than the intra-assay coefficient of variation of the analyzer in 43 of the 60 samples (72%). Utilizing the absolute values of the percentage change in the ACTH concentration compared to the reference samples in the analysis prevented statistical cancelation of the results that either increased or decreased. The horses that were included in the study were of ages relevant to the diagnosis of PPID, with horses ranging in ages from 11 to 27 years. Various breeds and sexes meant the group was an adequate representation of a general practice population of full-sized horses. If a larger number of horses were utilized, potentially more clinically significant effects resulting in misdiagnosis of PPID might have occurred. Although these changes in the immunoreactive ACTH concentration only caused 1 misdiagnosis within our study (7%), there is potential that the effects of stability could have a significant effect when monitoring ACTH concentrations in individual PPID horses over time, which is recommended for individual pergolide dosage adjustment. An erroneous result could be enough to alter treatment decisions.

Another limitation was that samples in general ambulatory practice might be exposed to a variety of temperatures over a short period of time. A sample could be exposed to summer environmental temperatures (20 to 45 °C) for a period until being placed in a polystyrene cooler box with ice blocks or a portable fridge (4 °C). If no cooling device is available, and the sample is left in the vehicle, it could then be exposed to vehicle temperatures up to 70 °C in regions that experience high environmental summer temperatures [17]. These variations in temperatures could potentially cause ACTH concentrations to be even more unpredictable then those found in this environmentally controlled study.

## 5. Conclusions

In conclusion, the stability of equine ACTH is affected by short-term exposure to a temperature of 70 °C. When ACTH variation is reduced by grouping horses with and without PPID, our results using absolute values suggest that samples should be kept at 4 °C to reflect the true ACTH concentration. Exposure to temperatures of up to 40 °C for 1 h can still provide an appropriate diagnosis of PPID in most cases, but ACTH concentration might change by up to 28%, and 40% of samples exposed to 70 °C will be unmeasurable. TRH stimulation does not appear to affect immunoreactive ACTH stability.

## Data Availability

The figures show all individual horse data in the scatter plots. Specific information regarding individual horses can be obtained from the corresponding author.
